# Association of childhood mental health and cognition with longitudinal patterns of cannabis problems in adolescence

**DOI:** 10.1017/S0033291725001175

**Published:** 2025-04-30

**Authors:** Rachel Lees Thorne, Lindsey A. Hines, Chloe Burke, Hannah J. Jones, Tom P. Freeman

**Affiliations:** 1 Addiction and Mental Health Group, University of Bath, Bath, UK; 2 Centre for Academic Mental Health, Population Health Sciences, Bristol Medical School, University of Bristol, Bristol, UK; 3 MRC Integrative Epidemiology Unit, Population Health Sciences, Bristol Medical School, University of Bristol, Bristol, UK; 4 National Institute for Health Research, Bristol Biomedical Research Centre, University Hospitals Bristol and Weston NHS Foundation Trust and the University of Bristol, Bristol, UK

**Keywords:** ALSPAC, Birth Cohort, Longitudinal data, Cannabis use, CAST, Latent class analysis, Epidemiology

## Abstract

**Background:**

Adolescence is a key developmental period associated with an increased risk of experiencing cannabis-related problems. Identifying modifiable risk factors prior to the onset of cannabis use could help inform preventative interventions.

**Method:**

Analysis nested within a UK prospective birth cohort study, the Avon Longitudinal Study of Parents and Children. Participants (*n* = 6,049) provided data on cannabis use and symptoms of cannabis problems using the Cannabis Abuse Screening Test at two or more time points between the ages of 15–24 years. Risk factors included internalizing and externalizing disorders assessed at age 10 years, and cognitive function assessed at age 8 years via short-term memory, emotion recognition, divided attention, and listening comprehension.

**Results:**

Participants were mostly female (59.1%) and white (95.73%). Five patterns of adolescent cannabis use problems were identified using longitudinal latent class analysis: stable-no problems (*n* = 5,157, 85%), early-onset high (*n* = 104, 2%), late-onset high (*n* = 153, 3%), early onset low (*n* = 348, 6%), and late-onset low (*n* = 287, 5%). In adjusted models, externalizing disorders were associated with early-onset high [RR, 95% CI: 2.82 (1.72, 4.63)], late-onset high [RR, 95% CI: 1.62 (1.02, 2.57)], and early-onset low [RR, 95% CI: 1.82 (1.30, 2.55)] compared to the stable-no problems class. Internalizing disorders were associated with late-onset low only [RR, 95% CI: .50 (.26, .96)], and short-term memory with late-onset high only [RR, 95% CI: 1.09 (1.01, 1.18) compared to the stable-no problems class.

**Conclusions:**

Childhood externalizing disorders were consistently associated with increased risk of problematic patterns of cannabis use over adolescence, particularly early-onset and high levels of problems.

## Introduction

Cannabis continues to be the most widely used internationally regulated drug worldwide (United Nations, 2022). Use is particularly prevalent in younger populations (European Monitoring Centre for Drugs and Drug Addiction, 2022), who are more vulnerable to developing problems related to their cannabis use than older users (Leung, Chan, Hides, & Hall, 2020). Though psychosocial treatment options have been developed to help with reducing use, most people with problematic cannabis use do not seek out professional treatment (Kerridge et al., 2018; Mongan et al., 2022; Wu, Zhu, Mannelli, & Swartz, 2017). Meanwhile, the efficacy of available psychosocial treatments for cannabis problems is modest, and long-term outcomes are unclear (Lees et al., 2021). The course of problematic cannabis use in adolescence is associated with later adult externalizing disorders and psychotic experiences (Kosty et al., 2017). Therefore, problematic cannabis use in adolescence represents a clear target for preventative action, to reduce public health burden and treatment need during a crucial developmental period. Determining childhood risk factors for the development of cannabis problems could help develop targeted preventative measures. Using longitudinal cohort data, groups at particularly high risk for sustained problematic use can be identified, as has been established in longitudinal studies of patterns of cannabis use (e.g. Hill, Shanahan, Costello, & Copeland, 2016; Taylor et al., 2017). Cohort data are advantageous for this approach as repeated assessment of cannabis problems allows for investigation of changes over time within and between individuals. Previous studies using data-driven approaches have found that concurrent and retrospectively assessed childhood mental health disorders such as ADHD and other externalizing disorders, as well as other substance use and male sex, are associated with particularly risky patterns of cannabis use over time (e.g. increasing severity, or stable-high severity; Kosty et al., 2017; Marmet, Studer, Wicki, & Gmel, 2021). However, risk factors in these previous studies were either measured retrospectively, or concurrently with cannabis use, therefore it is unclear whether reverse causality or recall bias may affect these results. Further, no previous investigation has looked at childhood cognition as a risk factor or adjusted for this in analyses. Current use of cannabis in adolescence is associated with poorer cognitive function (Gorey et al., 2019), and potential negative effects of cannabis use on cognitive function have been widely reported, particularly in people with problematic cannabis use (Broyd et al., 2016; Curran et al., 2016; Lovell et al., 2020). However, the causal direction of these relationships is not well understood. Most research conceptualizes poorer cognitive function as an outcome of cannabis use. Cannabis can dose-dependently impair cognitive performance, including working and episodic memory, due to a high density of cannabinoid receptors in brain regions associated with cognitive function such as the hippocampus and the prefrontal cortex (Curran et al., 2016). However, there is also some evidence that cognitive deficits may occur prior to the onset of cannabis use and may be associated with early-onset use in particular (Castellanos-Ryan et al., 2017). Birth cohort data provide a unique opportunity to investigate the temporal association between cognition and cannabis use.

Here, we present data from the Avon Longitudinal Study of Parents and Children (ALSPAC), a prospective birth cohort study with validated measures of mental health and cognition, which can help to address previous issues of temporality and increase the quality of evidence of associations between mental health, cognition, and cannabis problems during adolescence. Using the longitudinal data available in ALSPAC, we aimed to identify different patterns of cannabis use problems that occur between the ages of 15 and 24 years. Based on research indicating that cognitive deficits might be present before the onset of cannabis use (Castellanos-Ryan et al., 2017), we hypothesized that poorer cognitive performance in childhood would be associated with more persistent later cannabis problems across adolescence. Additionally, based on findings of childhood mental health disorders being associated with patterns of later cannabis problems (Kosty et al., 2017), we hypothesized that the presence of a mental health disorder in childhood would be associated with more persistent later cannabis problems across adolescence.

## Method

The analysis plan, including exposure, covariate, and outcome variables as well as hypotheses, was preregistered on the Open Science Framework prior to analysis.

### Study population

ALSPAC is a UK population-based birth cohort, the methods of which have previously been described in detail (Boyd et al., 2013; Fraser et al., 2013; Northstone et al., 2019). Pregnant women residing in the former Avon Health Authority in south-west England with an estimated due date between 1 April 1991 and 31 December 1992 were invited to take part, resulting in a cohort of 14,541 pregnancies and 13,988 children alive at 1 year of age. When the oldest children were approximately 7 years of age, an attempt was made to bolster the initial sample with eligible cases who had failed to join the study originally. The total sample size for analyses using any data collected after the age of 7 is therefore 15,447 pregnancies, resulting in 15,589 fetuses. Of these, 14,901 were alive at 1 year of age. Study participants have been followed up at regular intervals, with the latest available relevant data for the current analysis from the age 24 assessment. Participants with available data to estimate their cannabis problems at least two time points between the ages of 15 and 24 years were included in the analysis, resulting in a sample of *n* = 6,049. Of the excluded sample, *n* = 6,893 did not complete any assessments of their cannabis problems and *n* = 1,959 were excluded due to only completing one cannabis problems assessment. The authors assert that all procedures contributing to this work comply with the ethical standards of the relevant national and institutional committees on human experimentation and with the Helsinki Declaration of 1975, as revised in 2013. Initial consent to the study was provided by parents/guardians. Ongoing informed consent for the use of data collected via questionnaires and clinics was obtained from participants following the recommendations of the ALSPAC Ethics and Law Committee at the time. Initial ethical approval for the ALSPAC cohort study was given by the Bristol and Weston Health Authority (E1808), Southmead Health Authority (49/89), and Frenchay Health Authority (90/8). Further information on ethical approval at subsequent assessments can be found at: https://www.bristol.ac.uk/alspac/researchers/research-ethics/. The current project (B3812) received ethical approval from the ALSPAC Law and Ethics Committee and the Local Research Ethics Committees.

### Assessments

#### Cannabis problems

Cannabis problems were measured at age 15, 16, 18, 20, 22, and 24 using the Cannabis Abuse Screening Test (CAST), a short screening tool that has been validated against DSM diagnoses (Legleye, 2018; Legleye et al., 2013). This six-item measure assessed past-year frequency of problems related to cannabis use. Items relate to morning or lone cannabis use, problems with memory due to cannabis use, friends or family having concerns about use, unsuccessful quit or reduction attempts and experience of other problems e.g. arguments, fights or accidents due to cannabis use. Participants indicated that each symptom occurred never, rarely, from time to time, fairly often, or very often. A symptom was coded as ‘met’ if the participant responded with ‘from time to time’ or more frequently for the first two symptoms, and from a response of ‘rarely’ or more frequently for symptoms 3–6, based on the original reported scoring of the CAST (Legleye, Karila, Beck, & Reynaud, 2007; Legleye, Piontek, & Kraus, 2011). A three-level CAST score was generated for each participant, based on the number of symptoms met: 0 = no abuse, 1–3 = low abuse, and 4–6 = high abuse. Those with no lifetime/past 12-month cannabis abuse were coded as no abuse. A score of 4 or more has been validated as indicating a high risk of problematic cannabis use (Legleye et al., 2007). The inclusion of the low abuse category enabled us to account for variation in severity of cannabis problems (including low severity of problems emerging in adolescence) and characterize changes in the level of problems over the six assessments.

### Exposures

Early childhood exposures were chosen to minimize the likelihood of participants using cannabis concurrently and reduce the likelihood of reverse causality. Mental health was assessed by parent report at age 10, measured via the development and well-being assessment (DAWBA). DAWBA is a structured clinical interview to diagnose psychiatric disorders based on DSM-IV and ICD-10 criteria. A six-level ordinal-categorical variable was developed for DAWBA, based on the probability of each disorder (Goodman, Heiervang, Collishaw, & Goodman, 2011). The disorders included were oppositional defiant disorder (ODD), conduct disorder (CD), attention deficit hyperactivity disorder (ADHD), major depressive disorder (MDD), generalized anxiety disorder (GAD), and social phobia. The prevalence of each of the prespecified mental health disorders of interest varied; however, for some individual disorders, there was a very low prevalence when stratified across the outcome classes. In order to maximize statistical power and comply with ALSPAC’s data policies on minimum cell counts in analyses, we (i) created two composite mental health variables, representing internalizing disorders (GAD, social phobia, MDD) and externalizing disorders (ODD, CD and ADHD). We also (ii) coded the presence of each mental health disorder by scores in the top 3 DAWBA bands, a deviation from the preregistration that stated we would only use the top 2 bands, to enable a greater chance of identifying lower-level problems.

Data on cognitive function were taken from the age 8 clinic. Children were assessed using the Diagnostic Assessment of Non-Verbal Accuracy (DANVA) on a measure of facial emotion recognition ability (Nowicki & Duke, 1994). The outcome variable used was the number of errors (0–24). They also completed the Wechsler objective language dimensions, a measure of listening comprehension, the outcome of which is the number of correct responses (Rust, 1996). Attention was measured using the test of everyday attention for children (TEA-Ch), dividing attention task, using a decrement score calculated from performance on two versions of the task and time taken to complete the task (Robertson, Ward, Ridgeway, & Nimmo-Smith, 1996). Finally, short-term memory (non-word repetition) was assessed (Gathercole, Willis, Baddeley, & Emslie, 1994). The number of correctly repeated items (0–12) was used as the outcome.

The ALSPAC study website contains details of all the data that is available through a fully searchable data dictionary and variable search tool: http://www.bristol.ac.uk/alspac/researchers/our-data/. Data at the age 24 clinic were collected and managed using REDCap electronic data capture tools hosted at the University of Bristol (Harris et al., 2009). REDCap (Research Electronic Data Capture) is a secure, web-based software platform designed to support data capture for research studies.

### Covariates

Covariates used in the analysis were selected based on theoretical and observational evidence of their association with both the exposures and the outcome. These included child characteristics of sex (assigned at birth), ethnicity (either White or Black, Asian, other ethnic group, derived from maternal report of own and partners ethnic group), and socioeconomic status (parent highest level of occupation). Parental occupation data were available as a derived variable with five categories: (I) professional, (II) Managerial and technical, (III) skills (nonmanual and manual), (IV) Partly skilled, and (V) unskilled work. Other covariates included child IQ (total IQ score on the Weschler intelligence scale) and parental mental health disorders or substance use disorder (maternal report at child age 8 of ever experiencing a mental health disorder, drug addiction, or alcoholism).

### Other variables

We planned and preregistered a validation of latent class group differences to investigate whether the latent classes have identified meaningful subgroups. Given that cannabis use commonly co-occurs with alcohol and cigarette use (Agrawal, Budney, & Lynskey, 2012; Yurasek, Aston, & Metrik, 2017), and CUD is associated with other drug use disorders (Hasin et al., 2016), we compared groups on alcohol and cigarette use problems at age 20, assessed using the Alcohol Use Disorders Identification Test (AUDIT) and the Fagerstrom Test for Nicotine Dependence (FTND; items relate to cigarette use). We also compared groups on age of first cannabis use, given that this is associated with severity of CUD (Millar et al., 2021).

### Analysis plan

Patterns of cannabis problems from age 15 to 24 were identified using longitudinal latent class analysis (LLCA), in Mplus version 8. LLCA models were characterized by both within-person variation and between-person differences in cannabis problems. Latent classes are determined based on groupings of similar patterns of responses over time. To determine the optimal number of latent classes, the model was run repeatedly, increasing the number of latent classes each time, and the model fit was assessed. Model fit was assessed using the sample-size adjusted Bayesian information criterion (SSA-BIC), the bootstrap likelihood ratio test (BLRT), and the Lo–Mendell–Rubin (LMR). Further, we considered the proportion of individuals in each class, entropy, convergence, and the interpretability of the classes. The LLCA was run across different levels of missing data (4+ and all 6 measurements present), as a sensitivity analysis (see Supplementary Table S1). Childhood risk factors for class membership were estimated using multinomial logistic regression, with latent class membership as the outcome. Participants were assigned to a latent class group with the highest posterior class membership probability. Latent classes were treated as an observed categorical variable given high model entropy (Sinha, Calfee, & Delucchi, 2021), with the ‘stable-no problems’ class as the reference category in multinomial logistic regression models. The two composite mental health variables and each cognitive variable were entered into separate models as exposures. Finally, we compared the groups on alcohol and cigarette use disorders using logistic regression models with latent classes as the predictor variable (‘stable-no problems’ as the reference group). This analysis was selected to estimate and compare the odds of other drug use disorders across problem classes to validate the selected class solution.

### Missing data and imputation

Complete data for CAST (that is all 6 time points) were provided by 1,173 participants. Of those with 2 time points of CAST (*n* = 6,049), 48% had available data on all predictors and covariates. The analytic (*n* = 6,049) sample had a greater proportion of females (*X* ^2^ = 427.10, *p* < .001), fewer ethnic minority participants (*X* ^2^ = 15.21, *p* < .001), and higher socioeconomic status (*X* ^2^ = 155.71, *p* < .001) than those excluded due to missing CAST data (*n* = 9,596). As is common with birth cohort studies, there was missing data on the exposure variables and covariates, ranging from 0.17% to 26.45%, due to both drop-out rate, as well as missing measure and item-level data. To reduce bias, missing data on predictor and covariate variables were imputed up to the outcome data sample size, using chained equations with auxiliary variables in STATA, version 17.0. Potential auxiliary variables were assessed for their relationship with the observed variables using correlation and logistic regression to assess whether they predicted missingness on the exposure/covariate (Hardt, Herke, & Leonhart, 2012). Further details on the Imputation model can be found in Supplementary Material section ‘Imputation model’.

A two-staged procedure determined that 54 imputed datasets were required to generate accurate point estimates and standard errors, based on the fraction of missing information (von Hippel, 2020). The imputation model contained all analysis variables (including exposure, covariate and outcome). Moreover, where appropriate, auxiliary variables were identified and added to the imputation model for individual variables. The analysis model was fitted to each imputed dataset, and the results combined using Rubin’s rules (Little & Rubin, 2019). Proportions of imputed categorical variables and distribution of imputed continuous variables were checked against the observed data.

## Results

### Patterns of cannabis use problems (LLCA)

Of the 14,901 children alive at 1 year of age in ALSPAC, *n* = 6,059 provided data on at least two time points of CAST across ages 15–24. Table 1 shows sample characteristics, and Supplementary Table S2 shows characteristics across latent classes (see Supplementary Table S3 for sample characteristics in excluded and complete case groups).

**Table 1.** Sample characteristics in imputed data

Variable	Prevalence*N* ^a^ (%)/Mean (SD)	
Sex		
Male	2,471	(40.85)
Female	3,578	(59.15)
Ethnicity		
White	5,791	(95.73)
Black, Asian or other ethnic group	258	(4.27)
SES (Parent highest occupation level)		
I (Professional)	271	(4.49)
II (Managerial and Technical)	1,674	(27.68)
III (Skilled, nonmanual)	1,659	(27.42)
III (Skilled, manual)	1,520	(25.12)
IV (Partly skilled)	752	(12.43)
V (Unskilled)	173	(2.86)
Maternal mental health disorder^b^		
Had experienced	973	(16.08)
Had not experienced	5,076	(83.92)
Maternal addiction^b^		
Had experienced	54	(.90)
Had not experienced	5,995	(99.10)
Child IQ (age 8)	106.73	(17.89)
Internalizing disorder^c^		
Yes	460	(7.60)
No	5,589	(92.40)
Externalizing disorder^d^		
Yes	656	(10.85)
No	5,589	(92.40)
Cognition		
Short-term memory (number of words correctly repeated)	7.38	(3.11)
Divided attention (decrement score)	5.16	(17.11)
Emotion recognition (number of errors)	4.51	(3.11)
Listening comprehension (number of correct responses)	7.61	(2.33)

a *N* estimated from imputed proportions as data were incomplete.

b Maternal mental health and addiction assessed as presence versus absence of maternal self-reported experiences at child age 8.

c Internalizing disorder consists of GAD, social phobia, and depression. Mental health disorders inferred via top three bands of the DAWBA.

d Externalizing disorder consists of CD, ODD, and ADHD. Mental health disorders inferred via top three bands of the DAWBA.

**Figure 1.**
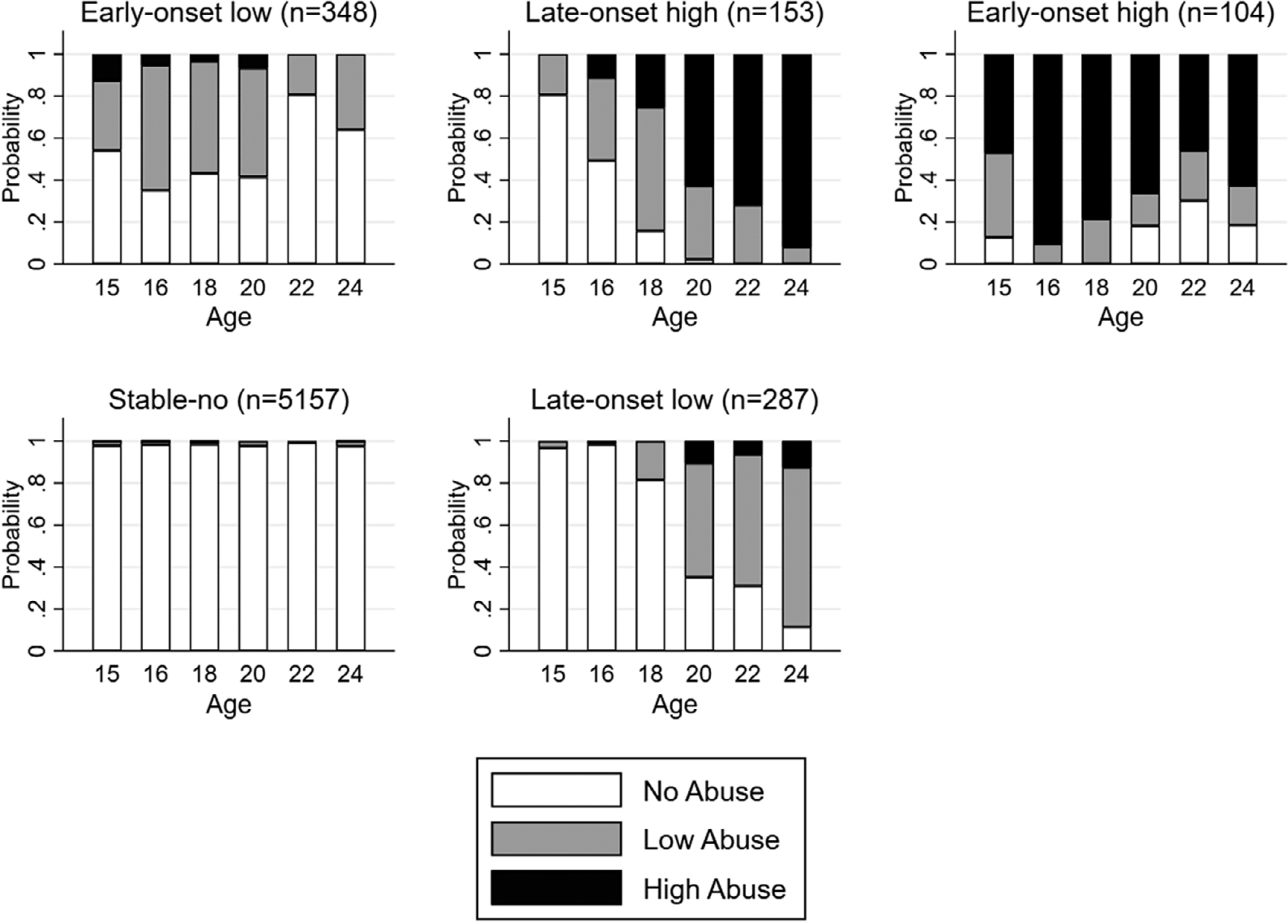
Data show the probability of no, low, and high abuse over the six-time points in each latent class.

A five-class solution was chosen as the best fit for the data, with the smallest SSA-BIC value and good interpretability of the classes compared to the 2-, 3-, 4-, and 6-class solutions (Table 2, see Supplementary Table S1 for model solutions with varying missing data). We identified a ‘stable-no problems’ class (*n* = 5,157, 85%), which included nonusers as well as users with very low likelihood of experiencing problematic use. The cannabis problem classes were defined as ‘early-onset high’ (*n* = 104, 2%), ‘late-onset high’ (*n* = 153, 3%), ‘early-onset low’ (*n* = 348, 6%), and ‘late-onset low’ (*n* = 287, 5%). See Figure 1 for a graphical representation of the five-class solution.

**Table 2.** Choosing the latent class solution

	Number of classes				
	2	3	4	5	6
2+ measures (*n* = 6,049)					
Number of observations/class count	1 = 746 (12%)	1 = 257 (4%)	1 = 193 (3%)	1 = 348 (6%)	1 = 85 (1%)
	2 = 5,303 (88%)	2 = 653 (11%)	2 = 334 (6%)	2 = 153 (3%)	2 = 324 (5%)
		3 = 5,139 (85%)	3 = 5,163 (85%)	3 = 104 (2%)	3 = 160 (3%)
			4 = 359 (6%)	4 = 5,157 (85%)	4 = 179 (3%)
				5 = 287 (5%)	5–4,989 (82%)
					6 = 312 (5%)
SSA-BIC	14,763.18	14,533.93	14,440.23	14,434.76	14,448.62
Entropy	.86	.83	.86	.86	.81
LMR *p*-value	<.001	<.001	<.001	.003	.320
BLRT *p*-value	<.001	<.001	<.001	<.001	<.001
Smallest class size	12%	4%	3%	2%	1%

### Predictors of patterns of cannabis use problems

Externalizing disorders were associated with greater than three times the likelihood of early-onset high problems (3.23, 95% CIs: 2.01, 5.21), twice the likelihood of late-onset high problems (2.16 95% CIs: 1.38, 3.38), and almost twice the likelihood of early-onset low problems (1.91 95% CIs: 1.38, 2.63). Adjusting for relevant covariates attenuated these relationships; however, externalizing disorders were still associated with almost three times the likelihood of early-onset high problems and almost twice the likelihood of early-onset low problems. There was little evidence that cognitive exposures as well as internalizing disorders were associated with later cannabis problem classes, although internalizing disorders were associated with a lower likelihood of late-onset low problems (.50, 95% CIs: 26, .96) and worse performance on short-term memory was associated with an increased risk of late-onset high problems (1.09, 95% CIs: 1.01, 1.18). See Table 3 for details of the adjusted and unadjusted multinomial regression models.

**Table 3.** Relative risk ratios from multinomial logistic regression models, each class compared to the ‘stable, no problems’ class

	Early-onset high (*n* = 104)		Late-onset high (*n* = 153)		Early-onset low (*n* = 348)		Late-onset low (*n* = 287)		Stable-no problems (*n* = 5,157)
	Unadjusted RR	Adjusted^a^ RR	Unadjusted RR	Adjusted^a^ RR	Unadjusted RR	Adjusted^a^ RR	Unadjusted RR	Adjusted^a^ RR	
Internalizing disorders	1.33 (.66, 2.68)	1.19 (.59, 2.43)	.69 (.32, 1.50)	.62 (.28, 1.38)	.91 (.58, 1.5)	.89 (.56, 1.41)	.50 (.26, .96)	.50 (.26, .96)	**1.0 (ref)**
Externalizing disorders	3.23 (2.01, 5.21)	2.82 (1.72, 4.63)	2.16 (1.38, 3.38)	1.62 (1.02, 2.57)	1.91 (1.38, 2.63)	1.82 (1.30, 2.55)	1.45 (1.00, 2.10)	1.30 (.89, 1.92)	**1.0 (ref)**
Short-term memory	1.02 (.94, 1.12)	1.00 (.90, 1.10)	1.07 (1.00, 1.15)	1.09 (1.01, 1.18)	1.04 (.99, 1.10)	1.03 (.97, 1.08)	1.05 (1.00, 1.11)	1.04 (.98, 1.10)	**1.0 (ref)**
Divided attention	1.00 (.98, 1.01)	1.00 (.98, 1.01)	1.01 (1.00, 1.01)	1.00 (.99, 1.01)	1.00 (.99, 1.01)	1.00 (.99, 1.01)	1.00 (.99, 1.01)	1.00 (.99, 1.01)	**1.0 (ref)**
Emotion recognition	.98 (.91, 1.07)	.99 (.91, 1.08)	.99 (.93, 1.06)	.98 (.91, 1.05)	.96 (.91, 1.01)	.96 (.91, 1.01)	.94 (.89, 1.00)	.94 (.89, 1.00	**1.0 (ref)**
Listening comprehension	1.08 (.97, 1.21)	1.04 (.93, 1.18)	1.05 (.96, 1.15)	1.04 (.94, 1.15)	1.02 (.96, 1.08)	.99 (.93, 1.06)	1.05 (.99, 1.13)	1.02 (.95, 1.10)	**1.0 (ref)**

a Adjusted for sex, ethnicity, IQ, socioeconomic status, maternal mental health disorder, and maternal addiction.

### Complete case analysis

As a sensitivity analysis, we repeated the multinomial regression analyses in the complete case data (*n* = 1,173). The pattern of effect sizes was similar across both sets of analyses. Full details of the complete case analysis are available in the Supplementary Table S4.

### Comparison of latent classes

To validate the latent classes, we investigated associations with alcohol use disorder and cigarette dependence at age 20 and on age of first cannabis use. As shown in Table 4, the pattern of cigarette dependence across the groups was similar to the severity of cannabis problems, with the greatest level of cigarette problems in the two classes characterized by high number of cannabis problems (early and late onset high abuse). Hazardous alcohol use showed a less consistent pattern compared to cigarette problems, with high levels of alcohol problems across all groups, and in the late-onset low group in particular.

**Table 4.** Comparison of latent classes against the stable-no problems group on alcohol and cigarette problems at age 20

	Hazardous alcohol use^a^				Cigarette dependence^b^				Age of first cannabis use	
	*N* (%)		OR^c^ (95% CIs)		*N* (%)		OR^c^ (95% CIs)		Mean (SD)	
Early-onset high	41	(74.55)	2.79	(1.52, 5.14)	13	(22.81)	12.44	(6.44, 24.01)	13.1	(1.25)
Late-onset high	64	(73.56)	2.65	(1.64, 4.29)	23	(25.56)	14.45	(8.56, 24.40)	14.5	(1.78)
Early-onset low	144	(70.59)	2.29	(1.68, 3.12)	23	(11.17)	5.29	(3.25, 8.62)	14.2	(1.70)
Late-onset low	146	(80.22)	3.87	(2.67, 5.61)	18	(9.33)	4.33	(2.54, 7.39)	16.0	(2.07)
Stable-no problems	1,639	(51.19)	1.00		79	(2.32)	1.00		16.7	(2.55)

a Alcohol Use Disorders Identification Test (AUDIT), scores of 8 and above classed as hazardous use.

b Fagerstrom Test of Nicotine Dependence (FTND), scores of 4 and above classed as cigarette dependence.

c Logistic regression models had stable-no problems as the reference group.

*Note*: There is missing data on AUDIT and FTND; therefore, cell counts/proportions will not add up to the total sample in each class.

### Age of first cannabis use

Across the full sample, *n* = 15 participants recorded that their first use of cannabis was below the age of 10. In order to determine whether this had any impact on the relationship between exposures and outcome classes, we re-ran multinomial regressions with this group of participants removed. Model coefficients and confidence intervals were very similar to the main model, as was the prevalence of internalizing and externalizing disorders in the sample. See Table 4 for the mean age of first reported cannabis use by latent class.

## Discussion

In a UK-based prospective birth cohort, we identified five latent classes representing different longitudinal patterns of cannabis problems across adolescence. The classes identified were early-onset high (*n* = 104, 2%), late-onset high (*n* = 153, 3%), early-onset low (*n* = 348, 6%), late-onset low (*n* = 287, 5%), and stable-no problems (*n* = 5,157, 85%). Regression analyses indicated that externalizing disorders in childhood are associated with both early-onset cannabis problems (early-onset high and early-onset low) and high levels of problems (early-onset high and late-onset high) and are particularly associated with the combination of both factors (early-onset high). Internalizing disorders in childhood were associated with a decreased risk of having late-onset low cannabis problems and were not associated with the other problem classes. Childhood cognition showed no strong evidence of association with cannabis problems in adolescence.

The findings build upon previous studies demonstrating a role of externalizing disorders in childhood with problematic later cannabis patterns (Hill et al., 2017; Kosty et al., 2017). The current study strengthens the quality of evidence due to the prospective assessment of childhood mental health prior to cannabis use onset. This highlights the importance of externalizing disorder as a potential target for intervention prior to a child’s first engagement with cannabis use to prevent subsequent longitudinal patterns of cannabis problems. Healthcare professionals, parents, and schools could use these findings to support young people and prevent progression to problematic cannabis use during adolescence. Internalizing disorders in childhood showed a weaker association with increased risk of later cannabis problems than externalizing disorders, in line with previous research (Kosty et al., 2017; Marmet et al., 2021). Internalizing disorders were in fact associated with a reduced risk of late-onset low cannabis problems, indicating that the nature of the mental health disorder experienced in childhood might influence later risk of cannabis problems. Externalizing disorders typically have a younger age of onset than internalizing disorders (Kessler et al., 2007); therefore the relationship between internalizing disorders and cannabis problems could emerge later in adolescence. Moreover, we did not consider the joint influence of either having both internalizing and externalizing disorders, or both a mental health disorder and problems with cognitive processing. It is likely that a combination of such risk factors would exacerbate the risk of later cannabis problems, which could be investigated in future research.

We also did not find strong evidence to suggest an important role of cognition as a predictor of later cannabis problems, with only a small effect of worse short-term memory on late-onset high. Cannabis use has been reliably associated with deficits in cognitive functioning; however, studies usually do not account for cognitive functioning prior to cannabis use. These study findings provide evidence to suggest that the previously noted association between cannabis and cognition could be due to cannabis use resulting in difficulties with cognition, rather than those with cognitive deficits being likely to later have problems with cannabis use. However, stronger evidence has been previously found for cannabis-associated deficits in performance on verbal learning and memory and emotion processing, with mixed findings for response inhibition and working memory (Broyd et al., 2016; Lovell et al., 2020; MacKenzie & Cservenka, 2023). The tasks administered to the ALSPAC cohort assessed relevant skills (short-term memory, emotion recognition, divided attention, listening comprehension), though it should be noted that findings may have differed had we employed tasks such as a verbal learning and memory task or other tasks of executive function abilities.

Additionally, our preregistered analysis plan included children’s IQ as a covariate to adjust for the impact of a child’s general intellectual functioning and isolate the specific effects of different facets of cognition and mental health on later cannabis problems. However, as IQ and cognition may be underpinned by overlapping processes, we also ran the cognitive models without this covariate. As reported in Supplementary Table S5, the pattern of results remained consistent, with only slight changes in confidence intervals. Of note, a relationship emerged for emotion recognition, indicating that a greater number of errors was associated with a reduced risk of being in the late-onset low group compared to the stable-no problems.

Comparison of the classes with other problem drug use showed a consistent pattern of increased cigarette dependence in the higher severity classes. This is consistent with evidence that cannabis users in the UK are likely to coadminister with tobacco (Hindocha, Brose, Walsh, & Cheeseman, 2021). Alcohol problems were consistently related to all classes relative to the stable-no group, with the highest levels of alcohol problems in the late-onset low group. This could indicate that the classes identified show some degree of specificity to cannabis use problems (relative to alcohol problems) rather than representing a general liability to problem drug use, yet greater similarity to cigarette dependence (as cannabis is typically coadministered with tobacco in the UK). Notably, levels of alcohol problems were high across all groups, with 51% of the stable-no group indicating hazardous levels of alcohol use.

This work should be considered in light of several limitations. We used a large dataset from a general population birth cohort, with cannabis problems assessed using a clinically validated self-report screening tool six times over a key developmental period in which cannabis problems may emerge. However, missing data may still be a cause of bias, given that only children who provided data on their cannabis use up to at least age 16 (due to the inclusion criteria of at least two CAST measurements) were included. Therefore, children who dropped out of the study around or after the time exposures were assessed are not represented here. However, additional analyses indicated that rates of mental health disorders in this subsample were similar to those in the wider ALSPAC sample. This indicates that attrition by adolescence has not led to an under-representation of individuals with childhood mental health disorders in this analysis. As evidenced in this study, attrition in the ALSPAC sample is related to ethnicity, sex, and socioeconomic status, and therefore, these results may not generalize to other settings and warrant replication. ALSPAC used an inclusive and flexible sampling strategy, attempting to contact all expectant mothers within the geographical region of Avon. However, the resulting ALSPAC sample was less ethnically diverse than a national sample estimate and had a low proportion of ethnic minority participants (Boyd et al., 2013), meaning that potentially important differences in the relationship between childhood mental health and adolescent cannabis use across ethnic groups may have been obscured in our study. Additionally, the available ethnicity data on the sample were two groupings of ‘White’ and ‘Black, Asian or other ethnic group’. However, treating these identities as singular, homogenous groups could obscure important interindividual differences within each of these categories, as research indicates that different ethnic groups might have different risk or protective factors surrounding cannabis use and the development of CUD (Montgomery, Dixon, & Mantey, 2022).

Further, the low prevalence of mental health disorders in childhood may decrease statistical power. We chose an early childhood assessment of mental health to minimize reverse causality, given a body of evidence indicating cannabis use increases the risk of other mental health disorders. Finally, due to small exposure group numbers, we used a lower threshold for categorizing mental health disorder presence than we had preregistered. This aided in increasing statistical power and was necessary to meet ALSPAC data reporting requirements. However, this might have resulted in a weakened association between disorder status and later cannabis problems. Finally, while the current study examined the role of childhood risk factors for later patterns of adolescent problematic cannabis use, future research could investigate the impact of cannabis problem patterns on later cognitive function and mental health (including psychosis) in adulthood.

To conclude, externalizing, but not internalizing, disorders in childhood appear to be a potential risk factor for developing cannabis problems in adolescence, which can include early-onset, high level problems. They may therefore represent a good target for clinical intervention in childhood to reduce the incidence and public health burden attributable to CUD.

## Supporting information

Lees Thorne et al. supplementary material Lees Thorne et al. supplementary material

## Supplementary material

The supplementary material for this article can be found at http://doi.org/10.1017/S0033291725001175.
