# Supplementary material for: Association of childhood mental health and cognition with longitudinal patterns of cannabis problems in adolescence
Source: Psychol Med. 2025 Apr 30;55:e129. doi: 10.1017/S0033291725001175 (PMC12094623; doi:10.1017/S0033291725001175)
Supplement: Lees Thorne et al. supplementary material [file S0033291725001175sup001.docx]

# Supplementary Materials

**Association of childhood mental health and cognition with longitudinal patterns of cannabis problems in adolescence**

**Imputation model**

As is common with birth cohort studies, there was missing data on the exposure variables and covariates, ranging from 0.17% (participant sex) to 26.45% (cognitive assessment of emotion recognition), due to both drop-out rate, as well as missing measure and item level data. In order to increase power and reduce bias in the analysis due to missing data, we imputed all missing values on exposure and covariate variables up to our outcome sample. Including auxiliary variables in the imputation model can help to improve the model and make the missing at random assumption more plausible (White et al., 2009). Good auxiliary variables are those that strongly correlate with the observed values of our exposure/covariates, and/or are predictive of missingness on those variables.

Given these criteria, we assessed several variables in ALSPAC to see if they were appropriate to use as auxiliary variables for imputation of missing values for exposure/ covariates. These variables all had theoretical reasons to relate to the exposure or covariates. For the exposure variables, these included earlier measurements of cognition and mental health. For covariates, these included index of multiple deprivation (for socioeconomic status) and maternal childhood trauma (for maternal ever mental health disorder/addiction).

Potential auxiliary variables were assessed for their relationship with the observed variables using correlation and logistic regression to assess whether they predicted missingness on the exposure/covariate. The final auxiliary variables used were Townsend deprivation and Index of multiple deprivation scores, maternal mental health measured at 12 weeks gestation, strengths and difficulties questionnaire conduct problems at age 6, generalised anxiety disorder and oppositional defiant disorder assessed using DAWBA at age 7, and maternal alcohol use in pregnancy.

Table S1. Choosing the latent class solution

|  | Number of classes | | | | |
| --- | --- | --- | --- | --- | --- |
|  | 2 | 3 | 4 | 5 | 6 |
|  |  | | | | |
| 3 + measures (n=4601) | | | | | |
| Number of observations/class count | 1 = 534 (11%)  2 – 4067 (88%) | 1 = 493 (10%)  2 = 3933 (85%)  3 = 175 (4%) | 1 = 244 (5%)  2 = 136 (3%)  4 = 261 (6%)  5 = 3960 (86%) | 1 = 246 (5%)  2 = 105 (2%)  3 = 74 (2%)  4 = 3960 (86%)  5 = 26 (5%) | 1 = 64 (1%)  2 = 242 (5%)  3 = 228 (5%)  4 = 110 (2%)  5 = 139 (3%)  6 = 3818 (83%) |
| SSA-BIC | 12290.92 | 12066.43 | 11977.13 | 11966.78 | 11978.08 |
| Entropy | 0.90 | 0.86 | 0.89 | 0.90 | 0.84 |
| LMR p-value | <.001 | <.001 | <.001 | .021 | .718 |
| BLRT p-value | <.001 | <.001 | <.001 | <.001 | <.001 |
| Smallest class size | 11% | 4% | 3% | 2% | 1% |
| Complete case (n= 1173) | | | | | |
| Number of observations/class count | 1 = 108 (9%)  2 = 1065 (91%) | 1 = 84 (7%)  2 = 30 (3%)  3 = 1059 (90%) | 1 = 17 (1%)  2 = 48 (4%)  3 = 58 (5%)  4 = 1050 (90%) | 1 = 17 (1%)  2 = 39 (3%)  3 = 1047 (89%)  4 = 51 (4%)  5 = 19 (2%) | 1 = 18 (2%)  2 = 1049 (89%)  3 = 39 (3%)  4 = 17 )1%)  5 = 44 (4%)  6 = 6 (0.5%) |
| SSA-BIC | 3026.43 | 3008.13 | 3013.93 | 3028.81 | 3050.16 |
| Entropy | 0.93 | 0.93 | 0.91 | 0.90 | 0.91 |
| LMR p-value | <.001 | .130 | .024 | 0.524 | 1.000 |
| BLRT p-value | <.001 | . <.001 | <.001 | <.001 | <.001 |
| Smallest class size | 9% | 3% | 1% | 1% | 0.5% |

SSA-BIC – sample-size adjusted Bayesian Information Criterion, smaller values indicate better model fit

Entropy – varies from 0-1; indicated likelihood of accuracy of the model defined classes. There is no universally accepted cut-off criteria, however above 0.8 is generally deemed to be acceptable

LMR –Lo-Mendell-Rubin test – compares the k_0_ class model with the k_-1_ class model. Significant p-values indicate improvement with the addition of an extra class

BLRT -Bootstrap Likelihood-Ratio Test – compares the k_0_ class model with the k_-1_ class model. Significant p-values indicate improvement with the addition of an extra class

Table S2. Sample characteristics of latent classes

| Variable |  | |  | | Latent class | |  | |  | |
| --- | --- | --- | --- | --- | --- | --- | --- | --- | --- | --- |
|  | Early-onset high (n=104)  N (%) / Mean (sd) | | Late-onset high (n=153)  N (%) / Mean (sd) | | Early-onset low (n=348)  N (%) / Mean (sd) | | Late-onset low (n=287)  N (%) / Mean (sd) | | Stable no problems (n=5157)  N (%) / Mean (sd) | |
| Sex |  | |  | |  | |  | |  | |
| Male | 57 | (55.34) | 109 | (71.24) | 153 | (43.97) | 158 | (55.05) | 1990 | (38.66) |
| Female | 46 | (44.66) | 44 | (28.76) | 195 | (54.03) | 129 | (44.95) | 3158 | (61.34) |
| Ethnicity |  | |  | |  | |  | |  | |
| White | 90 | (92.78) | 128 | (95.52) | 294 | (92.16) | 248 | (95.02) | 4489 | (96.17) |
| Black, Asian or other ethnic group | 7 | (7.22) | 6 | (4.48) | 25 | (7.84) | 13 | (4.98) | 179 | (3.83) |
| SES |  | |  | |  | |  | |  | |
| I-III (Professional, Managerial and Technical, Skilled, non-manual) | 54 | (58.06) | 69 | (53.08) | 193 | (62.87) | 164 | (63.32) | 2718 | (59.91) |
| III – V (Skilled, manual, Partly skilled, Unskilled) | 39 | (41.94) | 61 | (46.92) | 114 | (37.13) | 95 | (36.68) | 1819 | (40.09) |
| Maternal mental health disorder |  | |  | |  | |  | |  | |
| Had experienced | 22 | (27.85) | 31 | (26.05) | 56 | (20.07) | 43 | (19.11) | 596 | (14.69) |
| Had not experienced | 57 | (72.15) | 88 | (73.95) | 223 | (79.93) | 182 | (80.89) | 3462 | (85.31) |
| Child IQ (age 8) | 109.61 | (14.44) | 106.92 | (14.91) | 108.23 | (16.15) | 108.86 | (15.66) | 106.75 | (16.15) |
| Internalising disorder |  | |  | |  | |  | |  | |
| Yes | 8 | (9.20) | 6 | (5.04) | 19 | (6.74) | 9 | (3.77) | 335 | (7.94) |
| No | 79 | (90.80) | 113 | (94.96) | 263 | (93.26) | 230 | (96.23) | 3886 | (92.06) |
| Externalising disorder |  | |  | |  | |  | |  | |
| Yes | 23 | (26.74) | 23 | (18.70) | 47 | (16.67) | 32 | (13.22) | 413 | (9.74) |
| No | 63 | (73.26) | 100 | (81.30) | 235 | (83.33) | 210 | (86.78) | 3828 | (90.26) |
| Cognition |  | |  | |  | |  | |  | |
| Short term memory | 7.49 | (2.42) | 7.79 | (2.07) | 7.65 | (2.45) | 7.70 | (2.38) | 7.36 | (2.51) |
| Divided attention | 4.40 | (17.12) | 6.78 | (21.43) | 5.45 | (12.49) | 4.72 | (11.11) | 5.03 | (15.86) |
| Emotion recognition | 4.45 | (2.45) | 4.49 | (2.45) | 4.23 | (2.51) | 4.12 | (2.60) | 4.55 | (2.74) |
| Listening comprehension | 7.92 | (1.73) | 7.80 | (2.12) | 7.68 | (2.02) | 7.82 | (2.03) | 7.60 | (1.96) |

Note: Maternal addiction not shown here due to cell counts <5. SES categories collapsed into lower [I-III (non-manual)] and upper [III (manual) – V] due to low cell count

Table S3. Sample characteristics of non-imputed data for full ALSPAC sample, excluded sample and complete case sample

| Variable | 1 measurement (*n*=1,959)  (Excluded from sample) | 6 measurements (*n*=1,173)  (Complete case) | Full sample (*n*=15,645)  (Before inclusion criteria of 2+ CAST applied) |
| --- | --- | --- | --- |
|  | N (%) / Mean (sd) | N (%) / Mean (sd) | N (%) / Mean (sd) |
| Sex |  |  |  |
| Male | 1084 (55.48) | 385 (32.85) | 7688 (51.13) |
| Female | 870 (44.52) | 787 (67.15) | 7347 (48.87) |
| Ethnicity |  |  |  |
| White | 1509 (95.51) | 1060 (96.80) | 11520 (94.95) |
| Black, Asian or other ethnic group | 71 (4.49) | 35 (3.20) | 613 (5.05) |
| SES |  |  |  |
| I (Professional) | 38 (2.48) | 67 (6.20) | 376 (3.25) |
| II (Managerial and Technical) | 313 (20.46) | 338 (31.30) | 2632 (22.78) |
| III (Skilled, non-manual) | 400 (26.14) | 323 (29.91) | 2892 (25.03) |
| III (Skilled, manual) | 457 (29.87) | 229 (21.20) | 3341 (28.91) |
| IV (Partly skilled) | 239 (15.62) | 109 (10.09) | 1802 (15.59) |
| V (Unskilled) | 83 (5.42) | 14 (1.30) | 512 (4.43) |
| Maternal mental health disorder |  |  |  |
| Had experienced | 216 (19.60) | 155 (14.82) | 1329 (17.36) |
| Had not experienced | 886 (80.40) | 891 (85.18) | 6326 (82.64) |
| Maternal addiction |  |  |  |
| Had experienced | 14 (1.23) | 9 (0.85) | 67 (0.85) |
| Had not experienced | 1125 (98.77) | 1054 (99.15) | 7817 (99.15) |
| Child IQ (age 8) | 97.52 (56.56) | 110.32 (46.36) | 102.06 (21.65) |
| Internalising disorder |  |  |  |
| Yes | 106 (9.52) | 73 (6.22) | 659 (8.66) |
| No | 1007 (90.48) | 1011 (86.19) | 6949 (91.34) |
| Externalising disorder |  |  |  |
| Yes | 192 (17.08) | 76 (6.73) | 1046 (13.63) |
| No | 932 (82.92) | 1012 (93.27) | 6627 (86.38) |
| Cognition |  |  |  |
| Short term memory (number of words correctly repeated) | 6.80 (7.26) | 7.55 (6.92) | 7.23 (2.51) |
| Divided attention (decrement score) | 3.03 (68.22) | 1.29 (56.29) | 5.67 (16.10) |
| Emotion recognition (number of errors) | 3.60 (10.72) | 3.76 (9.34) | 3.87 (3.81) |
| Listening comprehension (number of correct responses) | 7.06 (5.97) | 7.82 (5.79) | 7.46 (2.00) |

Note: sample size will vary for each variable due to missing data and might not add up to the total sample size for each group.

Table S4. Complete case analysis multinomial regression models

|  | Early-onset high | | Late-onset high | | Early-onset low | | Late-onset low | |
| --- | --- | --- | --- | --- | --- | --- | --- | --- |
|  | Unadjusted RR | Adjusted^a^  RR | Unadjusted  RR | Adjusted^a^  RR | Unadjusted  RR | Adjusted^a^  RR | Unadjusted  RR | Adjusted^a^  RR |
| Internalising disorders  (n=4948) | 1.18 (.56, 2.45) | 1.24 (.52, 2.93) | .62 (.27, 1.41) | .46 (.14, 1.45) | .84 (.52, 1.35) | .87 (.49, 1.56) | .45 (.23, .89) | .39 (.16, .97) |
| Externalising disorders  (n=4974) | 3.38 (2.08, 5.51) | 2.43 (1.32, 4.48) | 2.13 (1.34, 3.40) | 2.00 (1.16, 3.46) | 1.85 (1.33, 2.58) | 1.45 (.94, 2.24) | 1.41 (.96, 2.08) | 1.24 (.77, 2.00) |
| Short term memory  (n=4804) | 1.02 (.94, 1.12) | 1.02 (.92, 1.13) | 1.07 (1.00, 1.16) | 1.09 (1.00, 1.20) | 1.05 (1.00, 1.10) | 1.02 (.96, 1.08) | 1.06 (1.00, 1.12) | 1.07 (1.00, 1.15) |
| Divided attention  (n=4586) | 1.00 (.98, 1.01) | 1.00 (.98, 1.02) | 1.01 (1.00, 1.01) | 1.01 (1.00, 1.02) | 1.00 (.99, 1.01) | 1.00 (1.00, 1.01) | 1.00 (.99, 1.01) | 1.00 (.98, 1.01) |
| Emotion recognition  (n=4448) | .99 (.91, 1.07) | 1.00 (.92, 1.10) | .99 (.93, 1.06) | .95 (.88, 1.03) | .96 (.91, 1.00) | .98 (.93, 1.03) | .94 (.89, .99) | .95 (.89, 1.01) |
| Listening comprehension  (n=4810) | 1.08 (.97. 1.21) | 1.04 (.93, 1.18) | 1.05 (.96, 1.15) | 1.04 (.94, 1.15) | 1.02 (.96, 1.09) | .99 (.93, 1.06) | 1.06 (.99, 1.13) | 1.02 (.95, 1.10) |

^a^Adjusted for sex, ethnicity, IQ, socioeconomic status, maternal mental health disorder and maternal addiction

|  | Early-onset high | | Late-onset high | | Early-onset low | | Late-onset low | |
| --- | --- | --- | --- | --- | --- | --- | --- | --- |
|  | Unadjusted RR | Adjusted^a^  RR | Unadjusted  RR | Adjusted^a^  RR | Unadjusted  RR | Adjusted^a^  RR | Unadjusted  RR | Adjusted^a^  RR |
| Short term memory | 1.02 (.94, 1.12) | 1.02 (.93, 1.11) | 1.07 (1.00, 1.16) | 1.08 (1.01, 1.16) | 1.05 (1.00, 1.10) | 1.03 (.98, 1.08) | 1.06 (1.00, 1.12) | 1.05 (.99, 1.11) |
| Divided attention | 1.00 (.98, 1.01) | 1.00 (.98, 1.01) | 1.01 (1.00, 1.01) | 1.00 (.99, 1.01) | 1.00 (.99, 1.01) | 1.00 (.99, 1.01) | 1.00 (.99, 1.01) | 1.00 (.99, 1.01) |
| Emotion recognition | .99 (.91, 1.07) | .98 (.90, 1.06) | .99 (.93, 1.06) | .98 (.92, 1.05) | .96 (.91, 1.00) | .96 (.91, 1.01) | .94 (.89, .99) | .94 (.88, .99) |
| Listening comprehension | 1.08 (.97. 1.21) | 1.07 (.96, 1.20) | 1.05 (.96, 1.15) | 1.04 (.95, 1.14) | 1.02 (.96, 1.09) | 1.00 (.94, 1.06) | 1.06 (.99, 1.13) | 1.04 (.97, 1.11) |

Table S5. Cognitive models with IQ removed as a covariate

^a^Adjusted for sex, ethnicity, socioeconomic status, maternal mental health disorder and maternal addiction
